# Transforming Mental Health Care in Moldova through Telemedicine

**DOI:** 10.1192/j.eurpsy.2025.798

**Published:** 2025-08-26

**Authors:** M. Bivol, C. Radislav

**Affiliations:** 1 Department of Mental Health, Medical Psychology and Psychotherapy, Nicolae Testemitanu State University of Medicine and Pharmacy of the Republic of Moldova, Chisinau, Moldova, Republic of

## Abstract

**Introduction:**

In Moldova, telemedicine has emerged as a pivotal intervention in addressing the persistent disparities in mental health care. Geographic isolation, coupled with socio-economic constraints, severely limits access to essential mental health services. The prevalence of mental health disorders is alarming, with approximately 17.34% of the population affected, particularly by anxiety and depression among vulnerable populations. This underscores the urgent need for innovative, scalable solutions. Telemedicine harnesses advanced technology to enhance the availability and continuity of mental health support, particularly in rural areas where traditional services are often inaccessible.

**Objectives:**

This study investigates the transformative potential of telemedicine in tackling the unique mental health challenges faced by the Moldovan population. It specifically examines how high rates of anxiety and depression, exacerbated by socio-political factors and historical trauma, can be addressed through telemedicine strategies tailored to the local context.

**Methods:**

A comprehensive literature review was conducted utilizing PubMed, focusing on studies and systematic reviews published since 2020. This analysis aimed to identify global best practices in telemedicine and evaluate their applicability within Moldova’s socio-cultural and economic landscape. The synthesis of these findings will inform future telemedicine initiatives aimed at improving mental health outcomes.

**Results:**

The findings indicate that telemedicine has significantly enhanced access to mental health services, facilitating timely interventions while reducing the stigma often associated with seeking help. The implementation of remote consultations during the COVID-19 pandemic has been particularly illuminating, demonstrating the effectiveness of telemedicine in maintaining continuity of care for individuals in remote areas who struggle to access traditional mental health services. Furthermore, telemedicine has led to significant reductions in treatment costs, thereby making mental health support more accessible to at-risk populations.

**Conclusions:**

As telemedicine solidifies its role within Moldova’s mental health framework, it promises to enhance public health outcomes and promote a more equitable healthcare environment. Future policy initiatives must prioritize the integration of telemedicine into routine mental health practices. This includes fostering partnerships between healthcare providers, policymakers, and community organizations to ensure that telemedicine solutions are both sustainable and tailored to meet the diverse needs of all patients, particularly those in underserved regions.

**Disclosure of Interest:**

None Declared

